# Discovering trends of social interaction behavior over time: An introduction to relational event modeling: Trends of social interaction

**DOI:** 10.3758/s13428-022-01821-8

**Published:** 2022-05-10

**Authors:** Marlyne Meijerink-Bosman, Mitja Back, Katharina Geukes, Roger Leenders, Joris Mulder

**Affiliations:** 1 Department of Methodology & Statistics, Tilburg University, Warandelaan 2, 5037 AB Tilburg, The Netherlands; 2 Department of Psychology, University of Münster, Münster, Germany; 3 Department of Organization Studies, Tilburg University, Tilburg, The Netherlands; 4 Jheronimus Academy of Data Science, ’s-Hertogenbosch, The Netherlands

**Keywords:** Longitudinal data, Relational event modeling, Personality, Social interaction dynamics, Social networks

## Abstract

Real-life social interactions occur in continuous time and are driven by complex mechanisms. Each interaction is not only affected by the characteristics of individuals or the environmental context but also by the history of interactions. The relational event framework provides a flexible approach to studying the mechanisms that drive how a sequence of social interactions evolves over time. This paper presents an introduction of this new statistical framework and two of its extensions for psychological researchers. The relational event framework is illustrated with an exemplary study on social interactions between freshmen students at the start of their new studies. We show how the framework can be used to study: (a) which predictors are important drivers of social interactions between freshmen students who start interacting at zero acquaintance; (b) how the effects of predictors change over time as acquaintance increases; and (c) the dynamics between the different settings in which students interact. Findings show that patterns of interaction developed early in the freshmen student network and remained relatively stable over time. Furthermore, clusters of interacting students formed quickly, and predominantly within a specific setting for interaction. Extraversion predicted rates of social interaction, and this effect was particularly pronounced on the weekends. These results illustrate how the relational event framework and its extensions can lead to new insights on social interactions and how they are affected both by the interacting individuals and the dynamic social environment.

## Introduction

Through social interactions, we build and maintain social relationships, express and adjust our personalities, exchange information, communicate feelings, and satisfy our fundamental needs for social belongingness and social achievements (Back, 2021; Bakan, 1966; Baumeister & Leary, 1995; Hogan, 1983). Social interactions are a key source of well-being (Kushlev et al., 2018; Lucas et al., 2008; Mueller et al., 2019; Sun et al., 2019), and this appears to hold quite universally. However, the way in which we engage in social interactions and the antecedents that drive us to engage in (specific) social interactions or not appear much less universal and highlight differences on both the individual and interpersonal level in social interaction behavior (see Back 2021; Echterhoff & Schmalbach 2018; Hopwood 2018; Sadler et al., 2011, for overviews). Conceptual models on the development of social relationships emphasize the key role of successive social interactions (Altman & Taylor, 1973; Back et al., 2011; Fehr, 2008; Knapp et al., 2014): People get to know each other and initiate and build social relationships through the flow of shared social interactions over time.

Longitudinal empirical approaches to understanding what drives us to engage in social interaction, to repeat (or not) previous social interactions over time, and the eagerness or speed by which we reach out to others for interaction (or by which we respond to invitations to interact) are, however, scarce (Geukes et al., 2019). Without such approaches it is difficult to develop a fine-grained understanding of how and why social interactions unfold over time. Specifically, there are three key domains of substantive research questions that are to date difficult to investigate given the lack of truly dynamic longitudinal approaches (see ; Back, 2021; Back & Vazire, 2015). First, we need to better understand social interaction processes by which interaction partners influence each other’s behavior and develop more or less intense forms of social relationships. Despite calls for a dynamic, process-oriented view on social interaction (Leenders et al., 2016; Back, 2021), the majority of research on social interaction is based on aggregated counts of social contacts, which provide a relatively static view of social interaction. This prevents us from understanding how important social interaction processes evolve and influence each other over time. Adopting a dynamic view on social interaction enables us to change focus from stable properties of social interaction (‘Are more extraverted individuals on average involved in more interactions?’) to discovering social interaction processes (‘Are, given their previous interactions with each other and other individuals until this time, more extraverted individuals more likely to interact together next?’).

Second, we need a more continuous understanding of social interaction processes across acquaintance levels. Most research examines either zero-acquaintance contexts (i.e., getting-to-know scenarios like first freshmen interactions, speed-dates) or short-term acquaintance contexts (e.g., interactions among students or within network groups) or long-term acquaintance contexts (e.g., interactions among friends or romantic partners). What is currently missing are continuous analyses across time, showing us when certain processes are particularly important and when exactly other processes start to kick-in. That is, we are required to examine questions of stability and change of the driving mechanisms underlying social interaction, including when, how, and why change occurs. These questions are, for example, especially interesting in the context of newly acquainted individuals and the role of personality differences for relationship development. Previous research suggests that how personality drives social interaction changes when individuals become acquainted with each other (Leckelt et al., 2015; Leckelt et al., 2020) but this has not yet been properly tested in a truly continuous fashion.

A third domain of key open questions pertains to the role of interaction settings for social interaction processes. Here, we refer with a ‘setting’ for social interaction to its environmental context, i.e., whether the same individuals interact at home, at work, at a party, etc. It is widely recognized that both characteristics of individuals (e.g., personality) and the environmental context (e.g., situational features; Rauthmann et al., 2014) have important effects on behavior. It is shown that while personality traits affect behaviors across many settings, an individual’s behavior in a specific setting is substantially dependent on the characteristics of the environmental context (Sherman et al., 2015). For example, extraverts behave more sociable in general, and people, and extraverts in particular, behave more sociable in leisure situations (Breil et al., 2019a). It has, however, not yet been investigated in how far and how interaction settings together with individual characteristics influence interaction dynamics, that is, interaction processes over time (e.g., ‘Is the effect of extraversion on the probability to interact next more or less emphasized in leisure settings compared to study-related settings?’) To develop a deeper understanding of how social interaction unfolds over time, we need to examine how various driving mechanisms affect social interactions across and within different settings.

Here, we argue that the challenges involved in tackling these three domains of open research questions can be met by making use of recent advances in both the collection and the analysis of dynamic interaction data. Recent technological advances have increased the possibilities to collect samples of naturally occurring social interactions (Kozlowski, 2015). For example, we may collect e-mail data to learn about patterns of digitally mediated communication between employees in an organization (Mulder & Leenders, 2019; Quintane & Carnabuci, 2016), or learn about real-life social interaction processes by recording naturally occurring social interactions by utilizing mobile phones (Geukes et al., 2019) or proximity sensors (Elmer & Stadtfeld, 2020). Rich data that contains detailed information on the flow of social interactions over time thus becomes increasing available. Pairing such data with data on traits and other characteristics of the individuals allows researchers to study what drives individuals to start, maintain, dissolve, and manage their social interactions over time and how others play a role in an individual’s interaction dynamic.

Following previous suggestions for a micro-analytic approach in which social interactions are observed and studied on a fine-grained timescale (Butts, 2008; 2009; Geukes et al., 2019; Kitts & Quintane, 2019; Kozlowski, 2015; Leenders et al., 2016), the current study proposes and illustrates how such data can be potentially analyzed using a fairly new analytic technique, called *“relational event models”*. As will be illustrated in the current paper, relational event models are especially suited to study how continuous social interaction data unfolds over time. First, since social interaction processes operate beyond the individual (Back, 2021; Geukes et al., 2019), observations are mutually dependent and assumptions of standard data analytic methods are violated. Relational event models, however, can take into account complex network dependencies. The researcher can utilize this functionality of relational event models to study how individuals’ embeddedness in the overall dynamic network of interactions influences their interaction patterns (e.g., the more two individuals interact with the same others, the more likely it may be for them to interact with each other). Second, the order and timing of social interactions may contain important information on the dynamics of social interaction processes (Butts, 2008; Leenders et al., 2016; Quintane et al., 2014). When we have continuous-time interaction data, relational event models enable researchers to utilize this information in the data and study which factors influence the rhythm and speed of social interaction and how what happens next is influenced by what happened previously. Thus, in sum, relational event-modeling approaches provide psychology researchers with the analytical tools to overcome the previously described challenges and develop from continuous-time interaction data a detailed understanding of how social interaction unfolds over time.

In this article, we introduce relational event modeling and illustrate how this statistical framework can be employed to gain important insights from continuous-time social interaction data. First, a general introduction of relational event modeling is provided. We illustrate that relational event models enable us to study what drives social interaction processes by providing an example analysis of the data from the CONNECT study (Geukes et al., 2019). The data consist of observations of the real-life social interactions between university freshmen at the start of their curriculum. Specifically, we illustrate how relational event models can be used to study how students’ personality traits, demographic characteristics, the kind of situations they are in, their joint interaction history and their embeddedness in the overall dynamic network of interactions affect the way in which they develop and maintain social interactions with the other freshmen in a new community. Second, at the beginning of the observation period, the freshmen students are not yet acquainted with each other. As the students get to know each other, it is to be expected that what drives the social interactions between them changes (Leckelt et al., 2015; Leckelt et al., 2020). We illustrate how the basic relational event modeling analysis can be extended with a so-called “moving window” approach to study how the drivers of social interaction processes in the CONNECT data change over time. Third, we may distinguish between two settings for social interaction that the freshmen students move between: a leisure setting (e.g., an interaction in a restaurant or at a party) and a study-related setting (e.g., an interaction during class or as part of a learning activity). We further extend the analysis and model the setting (leisure versus study-related) as a dependent variable to study how the drives of social interaction processes in the CONNECT data behave across different settings for social interaction. Finally, we conclude with a discussion of the analyses in this paper and provide some outlook on future applications of relational event modeling in psychological research.

## Modeling continuous-time social interaction data

Relational event models can analyze any type of continuous-time social interaction data that can be viewed as a so-called *relational event history* (Butts, 2008). The term “relational event history” refers to a sequence of successive social interactions between a set of individuals that contains information on *who* are involved in the interactions and the time (or order) *when* the interactions took place. See Table 1 for an example of an observed relational event history. Each row in Table 1 represents a so-called *relational event*, which is minimally defined as an interaction between two or more individuals at a specific point in time (Butts, 2008). Relational events can be, and often are, extended with more information on the social interaction, such as the “setting” for interaction or the “duration” of the interaction (see the rightmost two columns of Table 1).

**Table 1:** The first few social interactions observed between freshmen students at the beginning of their new studies in the CONNECT study (Geukes et al., 2019)

Time (min.)	Student 1	Student 2	Setting	Duration (min.)
1	Anne	Ben	Leisure	30
61	Anne	Chris	Leisure	20
121	Dan	Emma	Study-related	15
151	Ben	Dan	Study-related	300
…	…	…	…	…

For illustration purposes, student IDs are replaced by fictitious names

Relational event models are especially suited to model relational event history data. They enable researchers to study how the complex interplay of individuals’ characteristics, their environment, and their history of interaction influences the probability for future social interaction, thereby continuously updating the past. In recent years, a number of relational event modeling approaches have been introduced (Butts, 2008; de Nooy, 2011; Perry & Wolfe, 2013; Stadtfeld & Block, 2017). The current paper focuses on Butts’ (2008) relational event model (REM), which provides an especially flexible framework for modeling relational event history data. Many of the concepts that we describe in the current paper, however, also apply to other relational event modeling approaches. For a comparison between different approaches, we refer the interested reader to Quintane et al.(2014, pp. 28-30).

In a REM, the probability of relational events to occur at a certain point in time or in the sequence is modeled. The core of the REM is the event rate *λ*. At a given time *t*, the event rate determines both (a) *who* will interact next, and (b) *when* the next interaction will take place. Therefore, a so-called “risk set” must be defined. This risk set $\mathcal {R}(t)$ contains all the events that can potentially occur at time *t*. Often, it makes sense to define the risk set as all possible directed or undirected pairs (*s*, *r*) of individuals. For example, given *N* individuals and undirected pairs, the risk set consists of $\frac {N(N-1)}{2}$ relational events. In principle, the events in the risk set can potentially occur at any point in time. It is possible, however, that the size of the risk set varies over time. For example, if an individual is not available for interaction for a certain time-interval during the study period, the events with that individual should be excluded from the risk set for the time points *t* that fall within that interval. Hence, we can flexibly account for individuals’ availability for social interaction to accurately model the social interaction processes among a set of individuals (see also ; Quintane et al., 2014).

While the event rate for a pair of individuals is assumed to change over the course of the study period, the event rate is assumed to remain constant from the time of the current event until the time of the next event. Given this piecewise constant hazard assumption, the waiting time from the current event at time *t* until the next event follows an exponential distribution, where the rate parameter is the sum of all the event rates for the pairs at time *t*:

1 $${\Delta} t \sim \text{Exponential} \Bigg( \sum\limits_{\mathcal{R}(t)} \lambda(s,r,t) \Bigg).$$

Thus, higher event rates at time *t* decrease the expected time until the next relational event (compared to lower event rates at time *t*).

Under the piecewise constant hazard assumption, the probability that the next observed relational event at time *t* is of the pair (*i*, *j*) is equal to

2 $$P((i,j)|t) = \frac{\lambda(i,j,t)}{{\sum}_{\mathcal{R}(t)}\lambda(s,r,t)},$$

i.e., the event rate of the pair (*i*, *j*) relative to the event rates of all the pairs (*s*, *r*) in the risk set $\mathcal {R}$ at time *t*, including (*i*, *j*) (Butts, 2008). Thus, pairs with a higher event rate at time *t* are more likely to be observed next than pairs with a lower event rate at time *t*.

The REM enables researchers to study the predictors that explain how an observed relational event history evolves over time by modeling the event rate. The event rate is modeled as the outcome variable on which predictors are regressed through a log-linear function:

3 $$\log \lambda(s,r,t) = \sum\limits_{p}{{\upbeta}_{p} x_{p}(s,r,t)}.$$

Here, β_*p*_ refers to the model parameter that denotes the magnitude of the effect of predictor *x*_*p*_ on the event rate. In this article, we follow Butts (2008) and refer to these predictors as *statistics*, but they are also sometimes referred to as “sequential structural signatures (SSS)” in the literature (Leenders et al., 2016; Pilny et al., 2016). Statistics can encode both exogenous and endogenous predictors of the event rate. First, exogenous predictors refer to any kind of variable that is external to the relational event history itself, such as individuals’ personality traits, age, or gender, or the environmental context (e.g., whether interaction occurs in a leisure or study-related setting). By including exogenous predictors of the event rate in the model we can study research questions like ‘Are more extraverted pairs more likely to interact next?’ or ‘Are pairs more likely to interact next if they are similar in age or gender?’. Second, it is assumed that each event in the observed sequence depends on the history of events. This assumption allows us to model the events independently, conditional on the history of events. How an event depends on the past is summarized by the endogenous predictors that are included in the model to explain the event rate. Endogenous predictors summarize characteristics of past interactions (Leenders et al., 2016), e.g., the volume of past interactions for a given student pair or the number of interaction partners with whom both students in a given student pair have interacted in the past. By including endogenous predictors of the event rate in the model we can study potential important research questions related to social interaction processes, like ’Does the time between subsequent interactions decrease if individuals have interacted more together in the past?’, or, interacting an endogenous predictor with an exogenous predictor, ’Are less extraverted pairs more likely to interact together next if they have interacted more together in the past compared to more extravert pairs?’. Estimation of the model parameters β_*p*_ associated with the predictors allows us to make inferences about the effects that drive how the sequence of social interactions evolves over time. For example, a positive parameter estimate for the exogenous predictor ‘extraversion’ indicates a tendency for more extraverted pairs to start interactions at a higher rate than less extraverted pairs.

A REM can be fitted both when the exact time points for the relational events are considered (e.g., $t_{1} = 1,\ t_{2} = 61,\ t_{3} = 121,\ \dots$) and when only the order of the relational events in the sequence is known (e.g., $t_{1} < t_{2} < t_{3} < \dots$). In the first case, the full likelihood is used, while in the second case the full likelihood reduces to an ordinal likelihood (pp. 163-165 ; Butts, 2008). In case the exact time points are available, it is recommended to use the full likelihood because using the ordinal likelihood instead would result in a loss of information (Quintane et al., 2014). When the ordinal likelihood would be used in such cases, nothing can be inferred about time-related concepts such as the speeding up or slowing down of social interaction. An advantage of using the ordinal likelihood, however, is that a REM can still be fitted when the timing of relational events is only known up to the order of the events in the sequence and the exact time points are unavailable.

## Analysis I: The basic relational event model

### Data

We show how to use the REM by providing some exemplary analyses of the CONNECT data (Geukes et al., 2019). The CONNECT study is an extensive research project into the joint development of personality and social relationships among freshmen students. Participants were 126 freshmen students who enrolled for the bachelor study in psychology at a university in Germany. Part of this study is an experience-sampling observation of the social interactions between these freshmen students. Our aim is not to fully analyze this dataset, but to show how the statistical approach of the REM can be used to study topics of psychological interest. The CONNECT data used in the following analyses as well as the code for all analyses can be found at https://osf.io/xjbm7/.

**Fig. 1:**
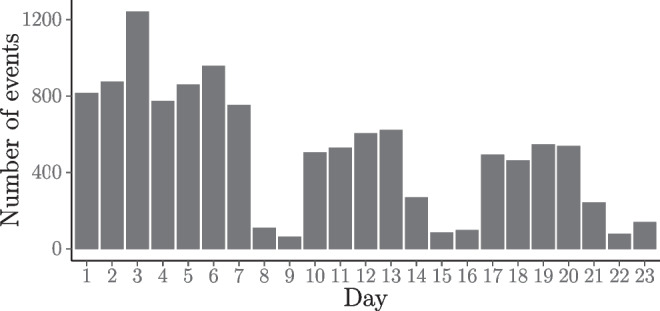
Frequency of observed relational events in the CONNECT study over the course of the observation period

Over the course of the first 23 days of their new studies, the participating freshmen students used an app on a smartphone to report every face-to-face interaction longer than 5 min as well as every digitally mediated interaction. See Table 1 for the first four observed relational events and Fig. 1 for the frequency of the observed events over the days. The specific time points for the relational events are defined in minutes relative to the onset of the observation period. The exact time points are available; thus we will use the full likelihood in our analysis. The relational events in the CONNECT study are undirected: we do not distinguish between sending and receiving students in a relational event. This means that, at any point in time, $\frac {126 \cdot 125}{2} = 7875$ potential relational events can occur among the 126 students.

### Theoretical background

Previous research indicates that extraverted individuals are more likely to select friends (Feiler and Kleinbaum, 2015; Selden & Goodie, 2018; Selfhout et al., 2010) and to have larger social networks (Wagner et al., 2014). Furthermore, extraversion is linked to a stronger motivation for affiliation in peer groups (Neel et al., 2016), and to more sociable behavior particularly in social interaction (e.g., ; Breil et al., 2019b). Extraverts also report to spend more time in social interactions (Asendorpf & Wilpers, 1998; Wilson et al., 2015). In general, extraversion most strongly relates to quantitative indicators of “getting ahead” in social groups such as the amount of social contact and social status (e.g., see Back 2021; Back & Vazire 2015; Grosz et al., 2020, for overviews). Agreeableness is shown to be related to being selected more as friend (Selden & Goodie, 2018; Selfhout et al., 2010). Similar to extraversion, agreeableness is linked to a stronger motivation for affiliation in peer groups (Neel et al., 2016). Agreeableness tends to be particularly related to qualitative indicators of “getting along” such as fewer social conflict (Asendorpf & Wilpers, 1998), particularly in long-term relationships, and less to the amount of social contact in newly emerging social relationships (e.g., see ; Back, 2021; Back & Vazire, 2015, for overviews). In the current analyses, we extend our understandings of the effects of the personality traits extraversion and agreeableness on social interaction behavior by studying their effects on the rate of continuously occurring social interactions. Specifically, we include personality traits (extraversion and agreeableness) to our model to understand how the personalities of two students affect the extent to which they choose each other as interaction partners.

Besides people’s personality traits (such as extraversion and agreeableness), another important human trait that affects behavior over time is habituation or routine–the tendency of humans to repeat past behavior (Leenders et al., 2016). Within the context of relational event models, this is often termed *inertia*. Inertia captures the tendency to repeat past interaction, and to repeat more those interactions that were more frequent in the past. In essence, inertia captures the routinization of social interaction choices (Leenders et al., 2016). Pilny et al., (2016) suggest that, following general theories of social networks, an inertia effect may be essential to include in any REM. A tendency for inertia is found in previous REM analyses of directed social interactions between students (Pilny et al., 2017; Stadtfeld and Block, 2017). Therefore, we add inertia to our model to study the tendency of the students in the CONNECT study to develop interaction routines and keep interaction with past partners.

Closure is the tendency of individuals to interact with others with whom they share past interaction partners, i.e., the friends of my friends become my friends (Leenders et al., 2016). The tendency for closure is often found to be an important feature in forming social networks (Robins, 2013). A tendency for closure goes beyond the pair and describes the social embedding of individuals in the larger network. Evidence for a tendency for closure is found in previous REM analysis of directed social interactions (phone calls) between students (Pilny et al., 2017; Stadtfeld & Block, 2017). There are several reasons why this might be expected (see also ; Leenders et al., 2016). Having communication partners in common can be the consequence of having similar preferences and behavior, which might make the students more attractive to each other (similarity attracts). Another driver can be that having joint communication partners increases the opportunity to meet or to learn about each other. Either way, we would expect that the closure or shared partner effect may be an important predictor of social interaction in the CONNECT study. We add a shared partner effect to our model to study whether students who interact with the same others are also quicker to interact among each other. A further question to explore is whether the effects of inertia and shared partners on the event rate act the same across students’ personality trait levels. In order to study this question, we add interactions between the endogenous mechanisms (‘inertia’ and ‘shared partners’) and students’ personality trait effects (‘extraversion’) and (‘agreeableness’) to our model.

Homophily, or the tendency to interact or form relationships with others who are similar on one or more features, such as sex or age, has found to be an important mechanism in forming social networks (McPherson et al., 2001; Snijders & Lomi, 2019). Previous research showed that demographic similarity, including having the same gender and age, positively predicts friendship formation among adolescents (Rivera et al., 2010; van Zalk & Denissen, 2015). It is therefore important to account for effects of gender and age similarity on the probability for students to interact in our analyses of the CONNECT data.

The students in the CONNECT study recorded their interactions with fellow students both during weekdays and weekends. However, from Fig. 1 we can see that the number of events between students is considerably and consistently higher on weekdays (when they have classes and other obligations) than the number of events on weekend days (when they are entirely free to do as they wish). Previous research among university students has found evidence for differences in communication patterns during weekdays compared to weekends (Masuda & Holme, 2019). Therefore, we introduce a weekend effect in our model to control for this difference in event rate and investigate if students interact differently during weekdays compared to weekend days. Finally, these freshmen regularly interact in groups, rather than in pairs. We will show a way to deal with that within the confines of the REM and analyze whether group interaction differs from dyadic interaction.

### Model specification

A typical relational event model includes characteristics of the individuals, of the pairs of individuals, and of the way they are embedded in the network at large. In our example below, we will include a selection of effects to study the research questions that we described above. Of course, many more kinds of variables are possible to analyze social interaction dynamics. For an overview, see, for example, Butts (2008), Leenders et al., (2016) and Vu et al., (2017).

In this first model, we assume that effects are constant over time. We will relax this assumption later.

#### Baseline

We include a baseline (intercept) effect to capture the baseline rate for starting social interactions. The baseline simply is a statistic that is always equal to 1 for every dyad. It plays the same role in the REM as an intercept in a linear regression model: it captures the average tendency of student pairs to start interactions when all other statistics are zero.

#### Gender similarity

Two statistics are used to summarize similarity in gender. First, the statistic *x*_both.male_(*s*, *r*) is equal to 1 if both students in the pair (*s*, *r*) are male and equal to 0 if not. Second, the statistic *x*_mixed.gender_(*s*, *r*) is equal to 1 if one student in the pair (*s*, *r*) is male and the other is female and equal to 0 if not. The student pair (*s*, *r*) in which both students are female acts as the reference category. In the CONNECT sample, the majority of the students is female (80%) and thus the majority of the potential student pairs (64%) consists of students who are both female. Student pairs of mixed gender make up 32% of the potential student pairs and the remaining 4% are student pairs where both students are male. A positive model parameter β_both.male_ would indicate that male student pairs (*s*, *r*) interact at a higher rate than student pairs with another gender composition. Similarly, a positive model parameter β_mixed.gender_ would indicate that mixed-gender student pairs (*s*, *r*) tend to interact at a higher rate than other student pairs. Comparing these effects helps analyze gender preferences in the developing of social interaction.

#### Age similarity

Based on previous research, we include similarity in age in our example model to test if it positively affects social interaction for freshmen students. One approach is to calculate the difference in age between students and use that difference as an explanatory variable in our model. In this specific dataset, age differences are, however, limited. When a population is quite homogeneous with respect to a personal characteristic like age, students who are older than the common age can be seen as “outsiders” and be interacted with differently—a phenomenon connected to surface-level diversity (Thatcher and Patel, 2012). Therefore, in this analysis we dichotomize the age of students in a “young” category (age 24 or younger) and a “comparatively old” category (age 25 or older). Two statistics are used to summarize similarity in age. The statistic *x*_both.older_(*s*, *r*) is equal to 1 if both students are aged 25 years or older and equal to 0 otherwise. The statistic *x*_mixed.age_(*s*, *r*) is equal to 1 if one student in the pair is “young” and the other is “older”. The student pair (*s*, *r*) in which both students are aged younger than 25 years acts as a reference category. The majority of the students in the CONNECT sample is classified as “young” (82%) and thus the majority of the potential student pairs (67%) consists of students that are both “young”. Student pairs of mixed age make up 30% of the potential student pairs and the remaining 3% are student pairs in which both students are “older”. A positive model parameter β_both.older_ would indicate that “older” student pairs are likely to interact at an event rate than student pairs with a different age composition. Similarly, a positive model parameter β_mixed.age_ would indicate that mixed age student pairs are likely to interact at a higher rate than student pairs with another age composition. This allows the researcher to discover any possible age-related faultlines and the tendency of older students to interact in a different manner from younger students (or mixed-age pairs).

#### Extraversion

Students in the CONNECT study provided self-report measures on personality traits by completing the GSOEP Big-Five Inventory (BFI-S; ; Hahn et al., 2012) that was part of an online survey. To obtain a measure of extraversion, the three BFI-S items that measure extraversion were averaged (*α* = .85). Responses to these items were allowed to range on a scale from 1 (does not apply at all) to 7 (applies perfectly). Almost all of the 126 students filled out these items. The responses for two students were missing and we replaced those by the group mean. The other 124 participants scored an average of 5.1 and a standard deviation of 1.1. Finally, the extraversion scores were standardized.

There are two ways in which a trait like extraversion can be included in an analysis. One approach is to include it as a fixed sender effect, where it can be assessed whether extraverted students start more interactions. However, since the dataset only includes undirected interactions (so we cannot distinguish whether the more extraverted person was the sender or receiver of specific interactions), we cannot show this here.

An alternative approach in the case of undirected pairs is to study whether interaction is driven by higher extraversion levels for the most extraverted student or least extraverted student in the pair. In particular, we want to study whether it takes a minimum level of extraversion to interact with others and whether overly extraverted students are attractive interaction partners or not. We do this by computing two extraversion scores for each student pair. The first measure is the minimum level of extraversion of the two students in a pair. The statistic *x*_extraversion.min_(*s*, *r*) is equal to the standardized extraversion score for the student with the lowest extraversion score in the pair (*s*, *r*). This statistic tells us that both students have *at least* a level of *x*_extraversion.min_(*s*, *r*) on extraversion. The higher this value, the extraverted the student pair can be considered to be. A positive model parameter β_extraversion.min_ would indicate that the higher the extraversion of the least extraverted student in the pair, the higher their interaction rate. So, if interacting with a (largely) unknown individual requires to have at least some minimum level of extraversion, this would follow from this analysis. This is a relevant question, considering that most students in the dataset were unfamiliar to each other at the beginning of the study.

Our second measure, *x*_extraversion.max_(*s*, *r*), is equal to the highest standardized extraversion score for the students in the pair (*s*, *r*). This captures an upper bound for extraversion: both students are not more extraverted than *x*_extraversion.max_(*s*, *r*). The lower this statistic, the less extraverted the students in the pair are. If we observe a positive model parameter β_extraversion.max_, this would indicate that student pairs with at least one highly extraverted member interact at a higher rate than pairs where both students are less extraverted.

#### Agreeableness

Students’ personality trait agreeableness is measured in the online survey with three BFI-S and two additional BFI statements (Rammstedt & John, 2007). Responds to these statements ranged from 1 (does not apply at all) to 7 (applies perfectly). To obtain a measure of agreeableness, the three BFI-S items and the two additional BFI items were averaged (*α* = .56). Again, two students did not fill out these items and their scores were replaced by the overall mean. Agreeableness had a mean of 5.0 and a standard deviation of 0.8. Finally, the agreeableness scores were standardized.

Similar to the extraversion measures we computed above, we define two agreeableness statistics. The statistic *x*_agreeableness.min_(*s*, *r*) is equal to the lowest standardized agreeableness score of the two students in the pair (*s*, *r*). This measure captures the minimal level of agreeableness of both students in a pair. A positive model parameter β_agreeableness.min_ would indicate that the higher the agreeableness of both students, the higher their interaction rate. Second, *x*_agreeableness.max_(*s*, *r*) is equal to the highest standardized agreeableness score for the students in the pair (*s*, *r*). This value shows that none of the students in the pair score higher on agreeableness than *x*_agreeableness.max_. A positive model parameter β_agreeableness.max_ would indicate that student pairs with higher levels of agreeableness interact at a higher rate than student pairs who both have lower agreeableness.

#### Inertia

It is common to add an inertia effect to a REM model by defining *x*_inertia_(*s*, *r*, *t*) as the (relative) number of previous (*s*, *r*) interactions at time *t*. The more past interactions between a pair of students, the more likely it is that this pair will interact soon again. The CONNECT data enable us to refine this measure and describe the intensity of past interactions between a student pair with greater detail. Because the CONNECT data include information on the exact starting and ending times of the interactions, we can also include the duration of past events into the measure. It is reasonable to expect that past events that lasted longer will be more likely to be repeated than brief past events. Therefore, we account for the duration of past interactions in the inertia effect. Moreover, the CONNECT dataset has information on a second feature that is likely to affect repetition: group interactions. A fair amount (36%) of interaction among the students occurs in groups of more than two students. It is reasonable to expect that being together with person A in a ten-person group will be a less strong trigger for repeated interaction with A than when the interaction with A occurred in a small group or with A directly. Hence, we let the number of students involved in a past group interaction affect the weight with which past relational events are added to the inertia count. Let *e* = {*t*_*e*_,*s*_*e*_,*r*_*e*_} refer to an observed relational event *e* at time *t*_*e*_ between students *s*_*e*_ and *r*_*e*_, let *A*_*e*_ refer to the set of students involved in the social interaction that relational event *e* was part of and let *d*_*e*_ refer to the duration of this interaction. We define the inertia statistic for the student pair (*s*, *r*) at time *t* as follows:

4 $$x_{\text{inertia}}(s,r,t) = \sum\limits_{t_{e} < t, s_{e} = s, r_{e} = r} \frac{1}{\vert A_{e} \vert - 1} \cdot \ln(d_{e}),$$

**Fig. 2:**
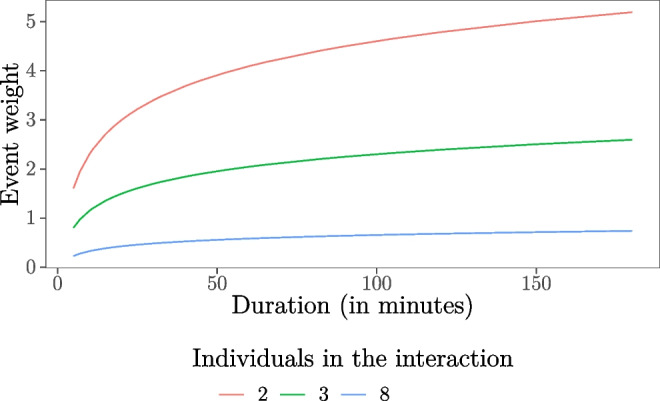
The weight with which past events between student pairs are included in the inertia count for increasing duration and interactions with two, three, or eight individuals

The measure *x*_inertia_(*s*, *r*, *t*) captures the sum of the past interactions between two students, weighted to the duration of the interactions and the number of students involved in each past interaction episode. To get an intuition of how this statistic weights the intensity of different kinds of past events between a student pair, Fig. 2 shows the weight of an event for increasing duration of interactions with two individuals (as in 64% of observed interactions), three individuals (as in 17% of observed interaction), or eight individuals (97.5% of the observed interactions is with eight students or less). The duration of the interactions ranges from 5 to 1805 min, the median duration is 30 min, and 97.5% of the interactions lasted 240 min or less.

In the case of endogenous statistics that are counts of past events, it is advisable to perform some kind of scaling method to make the statistic comparable over time and obtain well-behaved model parameters (Butts, 2008; DuBois et al., 2013; Schecter & Quintane, 2020). Here, we follow the recommendations of Schecter and Quintane (2020) and scale the weighted count by standardizing it per time point *t* as follows:

5 $$X_{\text{inertia}}(s,r,t) = \frac{X_{\text{inertia}}(s,r,t) - \bar{X}_{\text{inertia}}(t)}{SD(X_{\text{inertia}}(t))},$$

where $\bar {X}_{\text {inertia}}(t)$ and *SD*(*X*_inertia_(*t*)) refer to, respectively, the mean and standard deviation of the inertia statistic at time *t* over all pairs (*s*, *r*). A positive model parameter β_inertia_ indicates that student pairs (*s*, *r*) who interacted more intensively in the past are likely to interact at a higher rate in the future than student pairs who interacted less intensively in the past. Of course, a researcher does not have to include event duration or group size and can use the common unweighted measure if preferred.

#### Shared partners

The statistic *x*_shared.partners_(*s*, *r*, *t*) is the number students *h* that *s* and *r* both interacted with before time *t*. We standardize the variable per time point in the same way as for the inertia statistic (see Eq. 5). A positive model parameter β_shared.partners_ indicates that student pairs (*s*, *r*) who have more past shared partners are likely to interact at a higher rate in the future than student pairs who have fewer past communication partners in common. This statistic helps us understand whether having third parties involved (i.e., statistical significance of the coefficient) matters for the building up of relationships among freshmen and how strong the effect is (i.e., size of the coefficient). Finding a non-significant effect is informative as well, as that signals that interaction does not depend on shared others but is driven purely by individual or dyadic traits (depending, provided, of course, on the other variables and coefficients in the model as well).

#### Weekdays versus weekend

The statistic *x*_weekend_(*t*) is equal to 1 for all student pairs at time *t* if time *t* is in the weekend and equal to 0 if not. A negative model parameter β_weekend_ indicates that relational events in the weekend occur with at a lower rate in the weekend than interactions during the week.

#### Group interaction

None of the relational event models deal with group interaction in a fully natural way. Within the REM, there are two ways of dealing with group interactions. The first is to add potential groups as separate “actors” in the model (e.g., see ; Lerner et al., 2019), such that the individual actors can engage in interaction with these groups in addition to the interactions they can have with the other individuals (and even interactions between groups can be accommodated in this way). This is a useful method for relational events with a small set of actors, but can become computationally cumbersome for a large number of actors (and, hence, a larger set of potential groups). A possible refinement is to allow groups to come into existence (and dissolve) over the course of the observation period and include them as potential receivers during their existence (and exclude them when they are not active). This latter approach forms the basis of the recent DyNAM-i model of Hoffman et al., (2020). Although elegant, this latter approach focuses on the choice of individuals to join and leave a group and does not naturally address the situation when a group gets together (and dissolves itself) *as a group*. In the context of the freshmen, groups get together for study or for social activities and are more naturally seen as group action, rather than as interactions between a group and an individual.

In this paper, we show a simple alternative that appears somewhat artificial, but appears to work quite well nonetheless. The approach considers a group interaction as a set of interactions between all individuals in the group, occurring jointly and during the same time period. Mathematically, we divide group interactions (i.e., interactions with more than two students) into the set of dyadic interactions between every pair of students in the group happening in random order.^1^ Since relational events that are part of a group interaction have the same timestamp, which is not possible in a REM, a time difference between such relational events is induced before estimation (but after computation of the statistics, so the statistics are not affected by it). The time difference is such that these events are evenly spaced between the current time point minute *t* and the next minute *t* + 1. Since we induced a small time difference between relational events that were originally part of a group interaction, the rate of social interaction is artificially increased. We include a group effect in our REM to control for this artificial increase in the rate. The statistic *x*_group_(*t*) is equal to 1 for all student pairs in the risk set at time *t* if the relational event that we observe at time *t* is part of a group interaction and equal to 0 if not. Although somewhat artificial at first sight, this approach seems to work well in practice. It does make the underlying assumption that all students are aware of each other’s presence in the group and consider all other participants in the group as potential communication partners while in the group. This may not be realistic for very large groups or for groups that are externally regulated in their communication. However, the fast majority (81%) of the interactions in the CONNECT study occurs in small groups with only two or three students.

The inclusion of the “group” variable not only takes care of the inflated interaction rates during times of group interaction, but it also allows the researcher to study the behavior of individuals vis-a-vis a group context. For example, a researcher can study whether extraverted student pairs are more likely to interact within a group context. Or one can analyze whether social similarity (such as having similar age, similar gender, or similar shared partners) affects the tendency to interact within a group. Just by itself, a positive model parameter β_group_ indicates that relational events tend to occur more in a group setting than outside of groups.

#### Interaction effects

Similar to the inclusion of interaction effects between predictors in linear regression or loglinear regression, we can also include interaction terms between variables in a relational event model. Interacting the “inertia” and “shared partners” with the four personality trait effects results in eight interaction effects. A positive model parameter β_inertia.×.extraversion.min_, for example, would indicate that the effect of inertia on the rate of social interaction increases for student pairs with a higher minimum level of extraversion. As in standard regression models, the interpretation of interaction effects requires a researcher to also include the main effects into the model.

### Estimation

Appendix A provides the reader with the script for the preparation and estimation of the REM analysis. First, the statistics are computed using the novel R software package remstats.^2^ This software package has been developed to assist in the computation of commonly used REM statistics in an accessible manner. Second, estimation of the model parameters is realized using the R software package relevent (Butts, 2008). We build the models in a stepwise fashion, expanding the set of variables in consecutive steps. In total, we estimate five nested models (see Table 2).

**Table 2:** Relational event model parameter estimates with standard errors, BIC and goodness-of-fit (gof) results

Effect	Model 0	Model 1	Model 2	Model 3	Model 4
Baseline	–9.99 (0.01)*	–9.81 (0.01)*	–9.89 (0.01)*	–10.93 (0.02)*	–11.01 (0.02)*
*Personality trait effects*					
Extraversion min.		0.26 (0.01)*	0.20 (0.01)*	0.14 (0.01)*	0.11 (0.01)*
Extraversion max.		0.07 (0.01)*	0.06 (0.01)*	0.03 (0.01)*	0.00 (0.01)
Agreeableness min.		–0.02 (0.01)	0.00 (0.01)	0.01 (0.01)	0.02 (0.01)*
Agreeableness max.		–0.22 (0.01)*	–0.21 (0.01)*	–0.19 (0.01)*	–0.15 (0.01)*
*Endogenous effects*					
Inertia			0.12 (0.00)*	0.14 (0.00)*	0.32 (0.01)*
Shared partners			0.12 (0.00)*	0.11 (0.00)*	0.06 (0.00)*
*Demography effects*					
Both male				0.60 (0.03)*	0.55 (0.03)*
Mixed gender				–0.08 (0.02)*	–0.11 (0.02)*
Both older				0.17 (0.04)*	0.20 (0.04)*
Mixed age				–0.88 (0.02)*	–0.77 (0.02)*
*Event effects*					
Group				2.15 (0.02)*	2.11 (0.02)*
Weekend				–0.75 (0.02)*	–0.78 (0.02)*
*Interaction effects*					
Inertia × extraversion min.					0.03 (0.00)*
Inertia × extraversion max.					–0.09 (0.01)*
Inertia × agreeableness min.					0.10 (0.00)*
Inertia × agreeableness max.					–0.06 (0.00)*
Shared partners × extraversion min.					0.04 (0.00)*
Shared partners × extraversion max.					0.13 (0.00)*
Shared partners × agreeableness min.					–0.15 (0.01)*
Shared partners × agreeableness max.					0.01 (0.00)*
BIC	256931	256004	249036	236133	234520
gof	14.2%	14.3%	52.0%	54.7%	51.7%

### Results

#### Model selection and goodness-of-fit

The five models we fitted vary in their fit to the data and in the complexity of the models. A straightforward measure that balances fit and complexity is the Bayesian Information Criterion (BIC). This value can be computed directly from the maximum likelihood. Better models have lower BIC values. As can be seen from Table 2, Model 4 is the model with the lowest BIC, i.e., according to the BIC, the model with the best balance of fit and complexity among the five models.

To assess how well the models explain the observed relational event sequence, we perform a goodness-of-fit analysis. For each event, we calculate the predicted rates for each dyad (by plugging the coefficient estimates into Eq. 3). The probability of a specific dyad to host the next event is relative to its rate (see Eq. 2). This means that the model expects that it is most likely that the next event is going to occur among the dyads that have the highest predicted rates. If the model captures the empirical reality well, we would expect that in a fair proportion of the events, the actual event would occur for a dyad that was among the dyads with the highest predicted rates. Hence, we define goodness-of-fit (gof) as the proportion of instances in which the next observed event was in the top 5% of dyads with the highest predicted rates. Recall that at any point in time, 7875 events can potentially occur, but only one actually does. The model aims to predict which of these 7875 events occur at every point in time, which is an extremely ambitious objective. Hence, any model that somewhat consistently ranks the actual event among the top 5% of 7875 possibilities can be interpreted as performing really well. A similar approach to evaluate goodness-of-fit is performed by Pilny et al., (2016) and DuBois et al., (2013). For an in-depth goodness-of-fit analysis, we refer the interested reader to the approach proposed by Brandenberger (2019).

For relational events that were originally part of group interactions, we determine whether the highest ranked relational event within that group is in the top 5%. This gof metric thus refers to the ability of the models to predict the most plausible student pair who takes part in the next interaction. For the baseline-only, Model 0, this means that goodness-of-fit is calculated as 14.2%, i.e., we expect that if interaction occurs completely random, that in 14.2% of the interactions at least one student pair is correctly predicted (as being in the top 5%).

Goodness-of-fit results in Table 2 shows that introducing the personality traits in Model 1 only slightly increases the gof compared to the baseline-only Model 0. Note that the personality trait variables do not vary over time and thus predict the same student pairs in the top 5% for all time points. Apparently, these student pairs do interact slightly more often on average in the sequence than would be expected on random. Subsequently, there is a large increase in goodness-of-fit when the endogenous effects (inertia and shared partners) are introduced in Model 2. Model 2 is able to correctly predict (as being in the top 5%) at least one dyad in over half of all of the 2886 interactions over the course of the 3-week period. This large increase in goodness-of-fit for Model 2 indicates that these endogenous effects are very important in predicting the social interactions among freshmen students. Introducing the demography (gender and age) and event effects (group interactions, weekdays-versus-weekends) in Model 3 further increases the goodness-of-fit metric slightly. Introducing the interaction effects in Model 4 leads to a slight decrease in the goodness-of-fit metric. This indicates that interacting the endogenous effects (inertia and shared partners) with the personality traits extraversion and agreeableness has little value and even harms the predictive performance of our model. The best performing model (Model 3) correctly predicts (as being in the top 5 %) at least one student pair in almost 55 % of all interactions, which is remarkable and lends credence to the idea that these variables capture the most important drivers of the social interaction choices these freshmen made in the process of getting to know each other and new study mates.

#### Interpretation

Table 2 shows the estimated relational event model parameters and their standard errors for the five different models. Below, we interpret the parameters for the model with the best goodness-of-fit results, Model 3.

Because the relational event model is a loglinear model (see Eq. 3), we can take the log-inverse of the estimated model parameters to obtain a more meaningful metric for interpretation. For the baseline parameter the log-inverse refers to the average number of relational events per minute for a student pair with zeroes on all other statistics. After multiplication by the size of the risk set, we obtain the average predicted number of relational events per minute, $\exp ({\upbeta }_{\text {baseline}}) \times 7875 \approx 0.14$. The inverse of this number is the average expected number of minutes between two relational events: 7.08 min.

For all other effects, the log-inverses of the model parameters refer to baseline rate multipliers. For example, $\exp$ (β_inertia_) ≈ 1.15 indicates that for student pairs who interacted with one standard deviation more intensively in the past compared to student pairs who interacted with average intensity, the baseline rate of starting a social interaction is multiplied by 1.15. Thus, for these student pairs, the predicted waiting time between the start of two social interactions is on average $\frac {1}{\exp ({\upbeta }_{\text {baseline}}) \times 7875 \times \exp ({\upbeta }_{\text {inertia}})} \approx 6.17$ min.

**Fig. 3:**
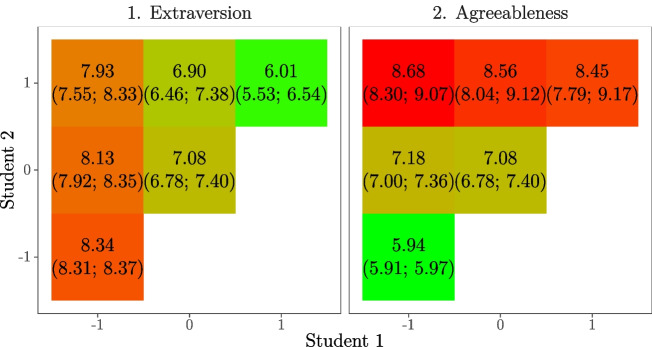
Expected time (in minutes and with 95% confidence interval) between interactions for different extraversion and agreeableness scores, based on the estimated model parameters for the personality trait effects in Model 4 (see Table 2). A score “0” refers to an average score on the trait, “1” refers to being 1 standard deviation above the mean and “-1” to 1 standard deviation below the mean. Comparisons of the rows informs us on the effect of the personality traits for the, respectively, most extraverted (β = 0.03, *p* < 0.05) or most agreeable student (β = − 0.19, *p* < 0.05) in the pair. Comparisons of the columns informs us on the effect of the personality traits for the, respectively, least extraverted (β = 0.14, *p* < 0.05) or least agreeable student (β = 0.01, *p* > 0.05) in the pair

Figure 3 summarizes how the personality traits affect the time between interactions for a student pair. Results in Table 2 show that, after controlling for all the other effects, higher extraversion levels for both the least and most extraverted student in the pair positively affect the event rate. Higher extraversion levels for the students in the pair are related to higher event rates. The expected time between subsequent interactions is most strongly determined by the least extraverted student in the pair, as visualized in Panel 1 of Fig. 3. The left column depicts student pairs where at least one member has an extraversion score of 1 standard deviation *below* the average. The cells in the table show the expected time until their next interaction. As can be seen, the extraversion of the most extraverted student in the pair has a small positive effect on the waiting time; the pairs with both students below the mean in extraversion tend to wait 0.41 min (5%) longer before they interact again than pairs with only one student below the mean and the other above the mean. The top row depicts student pairs where at least one member has an extraversion score of 1 standard deviation *above* the average. Here, we see that the extraversion of the least extraverted student in the pair has a larger positive effect on the waiting time; the pairs with one student above the mean in extraversion and the other below the mean tend to wait 1.92 min (32%) longer before they interact again that pairs with both students above the mean in extraversion. The results are subtle, but consistent: whereas the most extraverted communication partner has a small positive effect, the lowest extraverted partner most strongly determines the rhythm of social interaction.

Furthermore, results in Table 2 show a negative effect of agreeableness maximum and no effect of agreeableness minimum on the rate of interaction. As Panel 2 in Fig. 3 shows, after controlling for all of the other effects, when at least one partner scores 1 standard deviation *above* the mean in agreeableness (top row), the student pair takes longer to activate than student pairs where both students are low in agreeableness. Agreeableness is often considered to be a superordinate trait that includes compliance, modesty, and tender-mindedness (p. 217 ; Matsumoto and Juang, 2012). Considering that the participants do not know each other at the beginning of the study period and find themselves in new territory (new university environment, new city to live, new people to get to know, new tasks), highly agreeable individuals may be more conscientious and particular in their interactions, whereas low agreeable individuals might be more progressive and impulsive in their interaction choices.

The results in Table 2 further show that both endogenous variables (inertia and shared partners) have a positive effect on the rate of interaction. The students show clear signs of habituation and the development of “preferred” communication partners to continue interaction with repeatedly. The inertia parameter is 0.14, showing that, after controlling for all of the other effects, student pairs who interacted more intensively in the past are likely to interact at an even higher rate in the future. Similarly, student pairs with more past communication partners in common tend to interact at a higher event rate than student pairs who had fewer shared partners.

The positive parameter estimate for both-male indicates that, after controlling for all of the other effects, pairs of male students tend to have a higher rate of interaction than other student pairs. Conversely, the negative parameter for mixed-gender implies that male-female student pairs tend to have lower interaction rates than other student pairs. However, the majority of the CONNECT sample is female, such that 2525 pairs of students have mixed gender, 300 are all-male, and 5050 are all-female. Therefore, mixed-gender pairs have a higher a priori opportunity for interaction than all-male pairs. Indeed, the predicted time between interactions is on average $\frac {1}{300 \times \exp ({\upbeta }_{\text {baseline}}) \times \exp ({\upbeta }_{\text {both.male}})} \approx 102.01$ min for male-male pairs, $\frac {1}{2525 \times \exp ({\upbeta }_{\text {baseline}}) \times \exp ({\upbeta }_{\text {mixed.gender}})} \approx 24.00$ min for mixed-gender interactions, and $\frac {1}{5050 \times \exp ({\upbeta }_{\text {baseline}})} \approx 11.04$ min between female-female interactions. This shows that, despite the strong preference for same-gender interaction (and especially male-male interaction), interactions that involve one or two female students strongly outnumber interactions that are all-male.

In terms of the effect of age, Table 2 shows that, after controlling for all of the other effects, student pairs in whom both students are aged 25 years or older (“old”) display a higher expected rate of interaction than student pairs of another age composition. Student pairs of mixed age tend to interact at a lower rate than pairs of another age composition. This shows that there is a strong preference for same-age-group interaction (and especially for comparatively “old” student pairs). Like for gender, age is quite skewed, with 253 “old” dyads, 5253 “young” dyads, and 2369 dyads of mixed age. Hence, the expected time between interactions is, on average, $\frac {1}{253 \times \exp ({\upbeta }_{\text {baseline}}) \times \exp ({\upbeta }_{\text {both.older}})} \approx 186.85$ min for two older students, $\frac {1}{2369 \times \exp ({\upbeta }_{\text {baseline}}) \times \exp ({\upbeta }_{\text {mixed.age}})} \approx 56.66$ min for mixed-age students, and $\frac {1}{5253 \times \exp ({\upbeta }_{\text {baseline}})} \approx 10.62$ min for a pair of younger students.

The positive model parameter estimate for the “group” effect accommodates the increase in the event rate that was induced by dividing observed group interactions into dyadic relational events that follow each other rapidly. Considering that originally 2886 relational events were observed but 11,690 relational events after the division of group interactions into dyadic relational events, the event rate was increased by a factor $\log (\frac {11690}{2886}) \approx 1.40$. Subtracting this number from the estimated group effect, 2.15 − 1.40 = 0.75, gives us a “net” estimate of the tendency of freshmen to interact in pairs versus in groups. This positive effect indicates that, after controlling for all of the other effects, the freshmen engage in group interactions with a higher rate than in pairwise interactions.

The negative model parameter estimate for the “weekend” effect indicates a lower rate for engaging in social interactions during the weekend than during working days, after controlling for all of the other effects. On working days the predicted time between events is on average $\frac {1}{7875 \times \exp ({\upbeta }_{\text {baseline}})} = 7.08$ min and on weekend days the predicted time between events is on average $\frac {1}{7875 \times \exp ({\upbeta }_{\text {baseline}} + {\upbeta }_{\text {weekend}})} = 15.07$ min. This is in concert with Fig. 1 where the number of events on weekend days is considerably and consistently lower than the number of events on working days.

From this first example analysis, we can see that the relational event model can highlight the effect of personality and personal and interpersonal characteristics on how these adolescents interact in a natural experiment: a situation where the students are unfamiliar to each other and are stimulated to find attractive interaction partners. In itself, this is a very straightforward model—essentially just a loglinear model—but it not only allows us to uncover the drivers of how these youngsters learn to interact with each other, but the REM allows a researcher to quantify the effects in terms of time and timing as well: how much longer does it take between two individuals in condition A versus individuals in condition B?

## Analysis II: Relational event modeling with dynamic effects

In the analysis above, we made the underlying assumption that the effects are constant over the study period. However, the data concern freshmen who are starting a new life, with new people to get to know, a new place to live, and a new environment. As a result, we would expect to see some development of the way in which the freshmen develop their new persona as a student and learn whom to (not) interact with. Therefore, we now refine our model by dropping the assumption of constant parameter values and allow the parameters to vary over time. This allows us to study the second domain of key open questions outlined in the introduction, i.e., perform a continuous analysis of social interaction processes across acquaintance levels. Some of the interesting questions in this context are whether the effect of personality increases or decreases over time, how long it takes for inertia to kick in, or whether same-gender interaction may be considered a safe bet at the beginning of the period, while mixed-gender interaction gains attractiveness over time.

Our approach is to not put any constraints on the development of these effects (although that can certainly be done) and allow the parameter values to vary freely over the observation period. We do this by following Mulder and Leenders (2019) who extended the REM with a moving window approach. In this approach, a window of a pre-specified length slides over the entire observed relational event sequence. In each slice, the relational event model is fitted to the subset of relational events that falls within the window. Together, these slides create a picture of how the predictors of social interaction change over time. Following Mulder and Leenders (2019), a moving window REM can be fitted in the following steps:

1. Determine a window length.
2. Fit the specified REM to the subset of relational events that fall within the first window. Save the parameter estimates.
3. Move the window such that it partly overlaps with the previous window but also contains a new subset of relational events.
4. Fit the specified REM to the new subset of relational events. Save the parameter estimates.
5. Repeat Steps 3 and 4 until all relational events in the sequence are analyzed.

The choice of window length should depend on theoretical and statistical reasons. Ideally, the window length is chosen such that it corresponds to the empirically established or assumed temporal nature of the effects of interest (Mulder & Leenders, 2019). The smaller the window length, the more sensitive the results will be to each point in time (and the more estimates will reflect what happens on a given day or brief period of time). The wider the intervals, the smoother the development over time. Furthermore, the window length should be large enough such that it contains enough relational events to reliably estimate model parameters. The overlap between subsequent windows determines the smoothness of the results, where a higher number of events that overlap results in greater smoothness.

Since the interactions between the freshmen students in the CONNECT study are observed during the first three weeks of their acquaintance, we are interested to study how effects change over relatively short time intervals as the network develops during the getting-to-know-you processes. Therefore, we choose a window length of three days with two days overlap. This combination of window length and overlap between the windows allows us to study daily variation while also maintaining enough events in each window to reliably estimate model parameters. The number of events per window varies between 455 and 2937.

### Model specification

The script for the moving window analysis of the CONNECT relational event sequence can be found in Appendix B. To study how student interaction behavior develops over time in the CONNECT dataset, we apply the moving window approach to the same five models as analyzed in Section 4. However, it is no longer necessary to include a parameter to capture the difference in baseline event rate between the working days and the weekend; any weekday-weekend effect will be picked up automatically.

The endogenous statistics that were included in the model were slightly adapted to correspond to the expected dynamic nature of the social interaction processes in the CONNECT data. When interaction behavior is highly dynamic, it is important to consider how long past events influence future events (Brandes et al., 2009; Leenders et al., 2016; Mulder & Leenders, 2019; Quintane et al., 2013). Unfortunately, little theory exists in the literature to make an informed choice on how long past interactions influence future interaction behavior. Brandes et al., (2009) propose that the influence of past events decreases exponentially over time and that how fast this occurs depends on a half-life parameter. Quintane et al., (2013) specifically compare short-term and long-term time frames along which interaction processes may develop. Mulder and Leenders (2019) included only those past events in the computation of the endogenous statistics that occurred at most a fixed time period ago, corresponding to the nature of the moving window. Here, we follow the approach of Mulder and Leenders (2019), and let the influence of past events decrease corresponding to the expected dynamic nature of the social interaction processes in the CONNECT data. Consequently, we study patterns of interaction that develop over a relatively short time period.

### Results

#### Model selection and goodness-of-fit

**Fig. 4:**
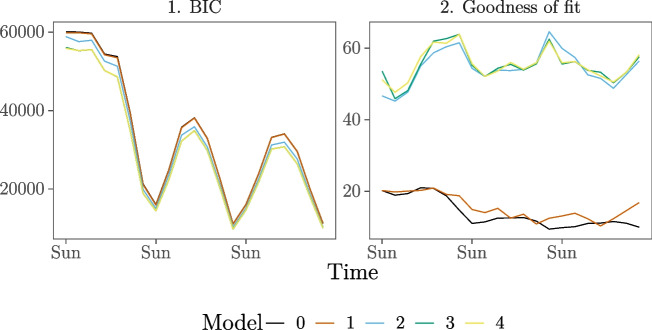
BIC and goodness-of-fit for the five models over time for the moving window REM

The results of the analyses are shown in Fig. 4. The BIC of Models 3 and 4 is consistently lower than that of the other models. Furthermore, we compute goodness-of-fit for the models over time in the same manner as before. Figure 4 shows that the goodness-of-fit drastically increases for the entire study period after inclusion of the endogenous effects (inertia and shared partners) in Model 2. Introducing the demography (gender and age) and event (group) effects in Model 3 and interaction effects in Model 4 on average slightly increase the goodness-of-fit further. Since Models 3 and 4 have consistently lower BIC values than the other models and are very similar in BIC and gof, we prefer the more parsimonious model of the two, Model 3, and will discuss that model’s results below. Model 3 has a fairly stable and high goodness-of-fit over the course of the study period, ranging between 45.8% and 63.8%. For two-third of the study period, the goodness-of-fit for Model 3 with the moving window applied is higher than for Model 3 in the basic REM analysis (which was 54.7%, see Table 2). Thus, even though the estimates in the moving window REM are based on fewer events (per window) than in the basic REM analysis (which includes all events for a single model fit), we can better predict the events that are likely to occur next with the moving window.

#### Interpretation

**Fig. 5:**
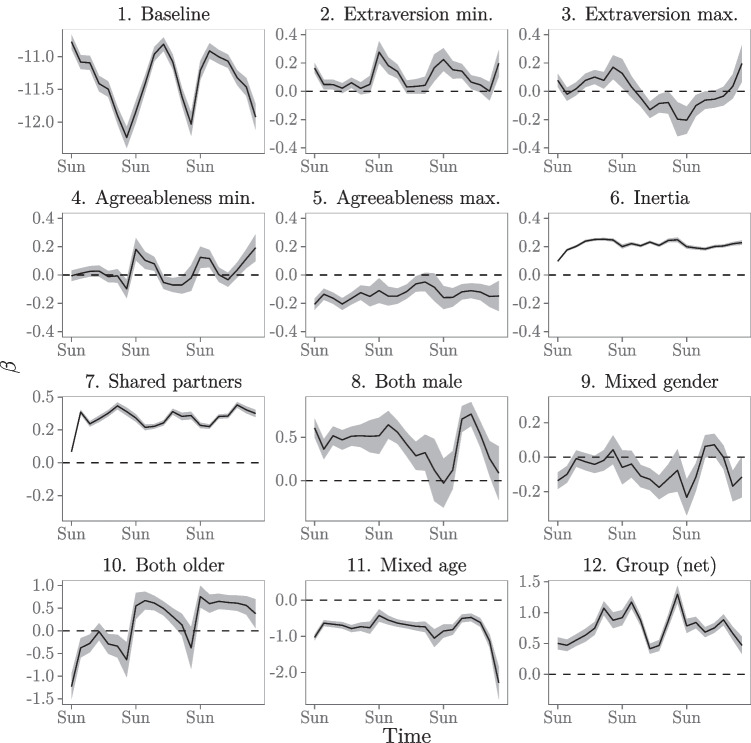
Dynamic effects on the rate of social interaction (Model 3)

Figure 5 shows how the effects on the rate of social interactions between freshmen students in the CONNECT study develop over time. Rather than interpreting every single effect, like we did above, we will highlight some interesting results. We can see from Panel 1 of Fig. 5 that the freshmen students tend to more actively interact during the weekdays than during the weekends (controlling for all other effects). In the previous analysis we also found this, but we do not need to estimate a separate parameter for this in the moving window model. This not only allows us to estimate a more parsimonious model, but it also allows us to detect a timing effect without having to expect and specify it beforehand. In our previous approach, we found a weekday-weekend effect because we included a parameter specifically for that as we expected such an effect on theoretical grounds. Alternatively, the moving window approach allows us to spot timing effects we might not have anticipated before specifying our model.

Panels 2 to 5 in Fig. 5 show the dynamic effects of students’ personality traits on how their social interactions develop over time. The effect of extraversion minimum clearly affects interaction during weekends (controlling for all other effects). During the week it does not really matter, but on weekends interaction is favored in dyads where both members are extraverted enough. Dyads where at least one of the students’ scores very low on extraversion have much less intense interaction than dyads where the least extraverted student is also fairly extraverted. This fits with the idea that interactions on weekends probably require more individual initiative than interactions on weekdays where students meet around educational activities. The effect of extraversion maximum appears to be positive during the first week: highly extraverted individuals are involved in interactions at higher rates than others. However, this turns around after the first week. It may be that extraversion helps in creating interactions in the first week, when students barely know anyone yet, but after that first week of getting acquainted other students become more active in interacting and the most extraverted individuals may even become less attractive communication partners during the weekdays.

For agreeableness, we observe a weekend effect: during the weekdays at the university agreeableness does not affect interaction rate, but on weekends, outside of the university environment, it helps to have at least fair level of agreeableness to be an attractive communication partner (or, to seek out other, more agreeable partners to hang out with). Throughout the observation period, there is no benefit to being very agreeable, as student pairs tend to be less intensive with highly agreeable individuals than with lower agreeable others. This may be connected to the more timid nature of highly agreeable persons, or simply to highly agreeable individuals to “go with the flow” and not push themselves as interaction partners. Of course, more in-depth research is needed to draw more informed conclusions about these effects.

Results in Panels 6 and 7 of Fig. 5 show that, after controlling for all other effects, the endogenous effects inertia and shared partners consistently positively affect the rate of social interaction throughout the observation period. Both these effects seem to develop in the first few days and remain relatively stable afterwards. These results suggest that such endogenous patterns of interactions develop early in a student network that starts at zero acquaintance. Moreover, the importance of these effects in explaining social interactions between freshmen students seems to remain relatively stable while acquaintance develops over time.

Results in Panel 8 of Fig. 5 suggest that, after controlling for all other effects, student pairs who are both male tend to interact at a higher rate than other student pairs, given their opportunity for interaction. This effect is relatively stable in the first two weeks, but, after two weeks, its effect seems to disappear during the weekends. Panel 10 shows that older students do not particularly seek each other out during the first week (possibly because they do not know who they are yet), but a preference towards connecting with each other does appear to develop after this first week of becoming acquainted. Age may be less of a trigger during weekends. Overall, there is a negative tendency of the different age groups to connect, this effect is quite stable throughout the study period.

Panel 12 of Fig. 5 shows the “net” group effect. Results show that connecting in a group context is very prevalent throughout the entire observation period.

Overall, the relational event model allows a researcher to draw conclusions of emergent behavior and how personality, demographics, social embeddedness, and human nature (i.e., human tendency towards habituation/inertia) drive how individuals interact and develop their social conduct. The moving window approach allows a researcher to not only study what the drivers are of the interaction choices these study participants make, but also uncovers how long it takes for the effects to kick in and for how long the effects then last. We believe this has the potential to add much detail to the development and refinement of theory of interpersonal human behavior.

## Analysis III: Relational event modeling with event types

In the models to this point, we consolidated the kinds of interaction the students could have into one. However, as outlined in the introduction, an important question associated with how social interaction unfolds over time is how various driving mechanisms affect social interaction across and within different settings. We now show a simple approach to address this question and check whether the variables we have found to drive social interactions between the students might actually have different effects for different kinds of interaction. This is done by including the setting for social interaction as an outcome variable in the analysis. In the CONNECT study, students report whether a given interaction occurred in a leisure or study-related setting. Letting *c* refer to the relational event type, Eq. 3 becomes:

6 $$\log \lambda(s,r,c,t) = \sum\limits_{p}{\upbeta}_{p} x_{p}(s,r,c,t)$$

Thus, the rate of social interaction for student pair (*s*, *r*) in setting *c* (leisure or work) is regressed on the set of model parameters β_*p*_ and statistics *x*_*p*_.

In the current example analysis, we can assume that every student pair is able to interact in either setting throughout the observation period. It is straightforward to alter the model if this were not the case. At every point in time, there are now $\frac {126 \times 125}{2}$ dyads × 2 settings = 15750 possible interactions among the 126 students and the two settings.

### Model specification

So far, not many studies have included event types to the dependent variable in their relational event modeling approach. Therefore, statistics that account for event types are limited in the literature and theory. In a study into the predictors of interpersonal communication in multi-team systems, Schecter (2017) defined several statistics that account for interaction types. Below, we suggest several statistics that draw some inspiration from Schecter’s work.

#### Study-related setting

We include a dummy *x*_study_(*s*, *r*, *c*) that is 1 if the potential relational event (*s*, *r*, *c*) is in a study-related setting and 0 if it is in a leisure setting. A positive model parameter β_study_ would indicate that student pair (*s*, *r*) is more likely to interact in a study-related setting than in a leisure setting.

#### Setting inertia

An interesting question regarding interaction dynamics is whether student pairs tend to keep interacting within the same setting or whether they tend to switch between settings. In other words: does leisure-based interaction trigger new leisure-based interaction, or does it tend to trigger work-related interaction instead? Therefore, we include an inertia effect that captures the intensity with which student pairs have previously interacted in a specific setting. The statistic for this effect is defined as

7 $$x_{\text{setting.inertia}}(s,r,c,t) = \sum\limits_{t_{e} < t \land s_{e} = s \land r_{e} = r \land c_{e} = c} \frac{1}{\vert A_{e} \vert - 1} \cdot \ln(d_{e}).$$

This statistic captures the intensity of all past relational events *e* between student pairs (*s*, *r*) in setting *c* at time *t*. The statistic is standardized for each time point *t*. A positive model parameter β_setting.inertia_ indicates that the more intensely student pairs (*s*, *r*) interacted before in setting *c*, the higher their interaction rates in the future in this setting.

#### Setting shared partners

When studying interaction across settings, it becomes of interest whether the effects are specific to a particular setting or consistent across all settings. For this purpose, we include a statistic *x*_setting.shared.partners_(*s*, *r*, *c*, *t*) that is equal to the number of shared interaction partners for students *s* and *r* within setting *c*. The statistic is standardized per time point. If this statistic is included in a model with a shared partners statistic, it captures whether the likelihood for a student pair (*s*, *r*) to interact in a specific setting *c* increases with their past interactions with shared partners in that same setting above whether future interactions rates are driven by their shared partners regardless of the setting. A positive model parameter β_setting.shared.partners_ indicates that shared partners in a specific interaction type stimulate student pairs to interact at higher rates in the future in this same setting.

#### Interaction effects

Student personality traits may have an effect on their preference to interact in specific settings. Therefore, we include interaction effects between the four personality trait effects and the study-related setting dummy. This allows a researcher to study if and how the effects of students’ personality traits differ between the two settings. Interacting the four personality trait effects with the study-related setting dummy results in four interaction effects. A positive model parameter β_study.×.extraversion.min_, for example, would indicate that the effect of the minimum bound of extraversion in student pairs increases the tendency to interact in a study-related setting compared to leisure interactions.

### Estimation

The script for the moving window REM analysis with event types for the CONNECT data can be found in Appendix C. We estimate three models (Models 3, 5, and 6), starting with the best model from the previous analyses (Model 3). In subsequent models (Models 5 and 6), we add setting effects as follows:

- Model 3: baseline, personality trait (extraversion and agreeableness), endogenous (“inertia” and “shared partners”), demography (age and gender) and event (“group”) effects.
- Model 5: baseline, personality trait (extraversion and agreeableness), endogenous (“inertia” and “shared partners”), demography (age and gender), event (“group”), study-related setting and setting endogenous effects.
- Model 6: baseline, personality trait (extraversion and agreeableness), endogenous (“inertia” and “shared partners”), demography (age and gender), event (“group”), study-related setting, setting endogenous and interaction (stud-related setting with personality traits) effects.

### Results

#### Model selection and goodness-of-fit

**Fig. 6:**
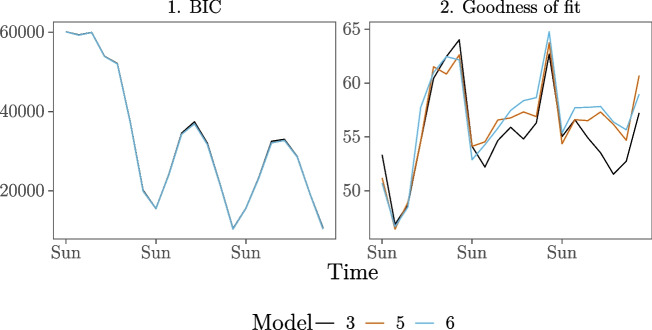
BIC and goodness-of-fit for the three models over time for the moving window REM with event types

Figure 6 shows that the three models have very similar BIC values overall. Models 5 and 6 have higher goodness-of-fit than Model 3, with Model 6 not showing real improvement in fit over Model 5. This indicates that there is little evidence for the value of the interaction effects between personality and setting. For all three models the goodness-of-fit remains fairly stable over time, with the fit improving after the first week. This may indicate the existence of some (external) factors that influence freshmen interacting with each other during the first week that are not yet in our model. The generally higher goodness-of-fit for Models 5 and 6 (with setting effects) compared to Model 3 (without setting effects) during the weekdays of the second and third week suggest that the setting effects are especially important in explaining the drivers of social interactions between the freshmen during the weekdays and less during the weekends.

#### Interpretation

**Fig. 7:**
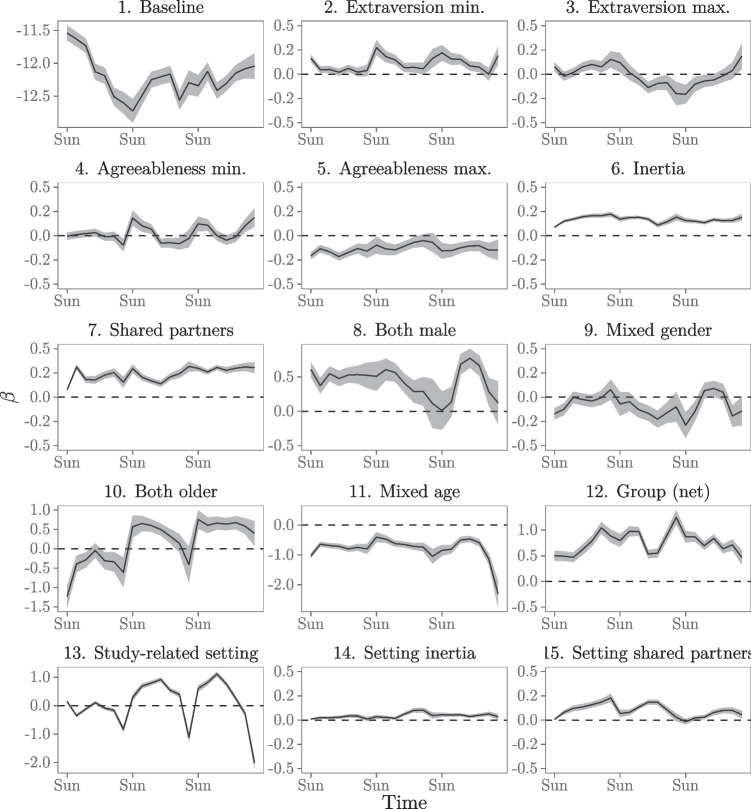
Dynamic effects on the rate of social interaction, considering the setting for interaction (Model 5)

Given that the BIC and goodness-of-fit results suggest an approximately equal fit for Models 5 and 6, we interpret the model parameters for the more parsimonious model of the two, Model 5. Figure 7 shows the estimated model parameters along the observation period. We focus our discussion here on the three setting effects that were newly introduced in this section, since the effects of the other variables are virtually identical to the previous model.

From Panel 13 of Fig. 7 we can see that there is a difference in the baseline tendencies for social interaction in a study-related and leisure setting (controlling for all other effects). During the first week, the students displayed no preference for interacting in one context of the other, but after that there is a clear tendency towards study-related interaction during the week and leisure-related interaction on the weekends. This is in itself not surprising (although students can also get together on weekends to work on class assignments or go on parties or hang-around during the week) and it shows that the relational event model can naturally pick this up. During the first week, it is to be expected that students are getting to know each other and switch a lot between study and leisure activities.

Results in Panel 14 of Fig. 7 show that, after controlling for all other effects, the freshmen display a small preference to repeat interacting in a specific setting, this effect stays consistently positive and small throughout the entire study.

Results in Panels 7 and 15 of Fig. 7 show that, after controlling for all other effects, student pairs with more shared partners are likely to interact at higher rates in the future than student pairs with fewer shared partners. This effect is enhanced by the setting in which the interactions with these shared partners occurred. Essentially, the more they studied with the same others in the past, the more they tend to study with each other in the future. Similarly, the more students engage in leisure activities with the same others in the past, the more they tend to do the same together in the future. While the shared partners effect suggests a tendency for clusters of students to form within the freshmen student network, the interesting implication is that these clusters appear to form especially within specific interaction contexts. This context-specific clustering seems to disappear during the weekends, because these are strongly leisure-driven for all students.

Together, this extension of the model shows that the dynamics and evolution of interactions among the freshman is affected by the context of the interaction and that the personality traits extraversion and agreeableness do not seem to interact with this.

## Discussion

With recent technological advances assisting real-life data assessment, relational event history data becomes increasingly available. This type of data has the potential to provide researchers with fine-grained information on social interaction dynamics and their role in social relationships and personality development (Back, 2021; Back et al., 2011; Bleidorn et al., 2020; Geukes et al., 2019). In this paper, we showed how such fine-grained social interaction information can be fruitfully analyzed making use of the REM modeling framework.

### Illustrative REM effects in the CONNECT data

The REM framework was illustrated using an experience-sampling study on social interactions between freshmen students who start interacting at zero acquaintance. A basic REM analysis provided us with insights on how predictors affected the rate of social interactions between these freshmen students on average over the entire observed event sequence. The analysis showed that the rate of social interaction among the freshmen students was influenced by a combination of demographic similarities, students’ personality traits, and endogenous effects. Regarding the latter, results underscore the relevance of including the history of social interactions as well as the broader social network (e.g., see ; Butts, 2008; Kitts & Quintane, 2019; Leenders et al., 2016) when trying to predict the occurrence of dyadic social interactions. Student pairs were more likely to interact in the future if they interacted more with each other in the past and if they had more past interaction partners in common. Results also point at the relevance of socio-demographic differences even within a highly selective and homogeneous sample of psychology students. Similarity in gender and age predicted the propensity to interact. Regarding the personality effects, results are in line with previously shown robust effects for extraversion (extraverts interact more) and more nuanced findings for agreeableness (e.g., see ; Back, 2021; Back & Vazire, 2015, for overviews). While agreeableness relates to better relationship quality (e.g., Asendorpf & Wilpers, 1998), it does not necessarily relate to a higher amount of social interaction. As shown in the present illustrative analyses, it can even go along with fewer social interactions. The REM allowed to investigate these basic effects of socio-demographic and personality within a joint framework, thereby controlling for the role of endogenous social interaction history effects and directly considering the timing of interaction events.

An extension of the basic REM framework with the moving window approach (Mulder & Leenders, 2019) allowed us to study how the role of predictors of the rate of social interaction changed over time. This is especially useful in situations where it is not realistic to assume that what drives social interaction is stable for the entire observed event sequence or when one is specifically interested in studying how the dynamics of social interactions unfold and change over time. This may not always be the case in lab-based studies, but this experience-sampling example study covered a 3-week period, which allows a researcher to study dynamics over an extended period of time and uncover how effects emerge, disappear, or show a rhythm (such as the weekend effect, where interactions were governed by other dynamics than weekday interaction). By applying the moving-window REM in a second example analysis, we found that personality trait effects changed after 1 week of becoming acquainted with each other. The effects of extraversion and agreeableness on the rate of social interaction operated differently between working days and weekends, which was a stable pattern across the study period. Similarly, socio-demographic effects on the rate of social interaction changed after freshmen became more acquainted and results suggested a trend in which these effects operated differently between working days and weekends. However, this is only an example analysis and more research is warranted to study further details of these trends and whether they continue after the third week of acquaintance. Results from our study also showed that the tendency to repeat past behavior (“inertia”) as well as the tendency to prefer interactions with those with whom one has many past communication partners in common (“shared partners”) developed already in the early stages of acquaintance and remained relatively stable as acquaintance developed.

Social interactions are not only characterized by the time at which they occur and who is involved but also by other important features, that is, the sentiment, mode of communication, setting for interaction, and so forth. The REM framework allows us to differentiate between types of events to study what drives social interactions of different type and how they dynamically affect each other over time. For example, two important settings for social interactions between freshmen students are a study-related setting and a leisure setting. By differentiating between these settings in our exemplary study in a third analysis, we found that freshmen students have a tendency to interact more within a setting if they have interacted more intensively in the past within this setting. This tendency was small, but constant over time. Moreover, findings indicate that clusters of interacting students tended to form within a setting.

The current exemplary application of the REM approach to study real-life social interactions was limited to one specific relationship type, age-group and cultural context (peer-relations among fellow students in Germany) and to an illustrative analysis of selected person- and context-level predictors. The REM approach we outlined, is, however, extremely flexible and can be used to examine all sorts of social interaction dynamics including social interactions with friends, colleagues and clients, family members, team members, and romantic partners in both early and later stages of relationship development. In doing so, future research should test and explore the role of a range of further individual (e.g., attachment styles, leisure preferences, values) and contextual characteristics (e.g., face-to-face versus computer-mediated interaction).

### Statistical considerations

Since the goal of the current paper was to provide an introduction into relational event modeling for psychology researchers, we had to make choices about the complexity of the analyses. Therefore, we choose a relatively simple solution to deal with group interactions. In this solution, all actors in an observed group were combined into all possible pairs. Since the goodness of fit results showed a remarkable recovery rate of about 55%, we are confident that this solution works well for the current data set. It should be noted, however, that other solutions exist that may be more appropriate in the case of modeling relational event history data with group events, see for example Hoffman et al., (2020) or Lerner et al., (2019). These approaches make different assumptions about how groups come to their existence. In Hoffman et al., (2020) actors join and leave groups one for one, while in Lerner et al., (2019) groups exists as entities that can also interact with each other.

Current studies in the literature that apply the relational event model mostly focus on directed relational events. Our exemplary analyses of the CONNECT data showcase that the flexibility of the REM framework is not limited to directed events, but can also handle undirected events. This is important because relational event histories with undirected events are commonly observed. For example, in recent years, wearable sensors are developed that allow the automated data collection of undirected face-to-face contacts (Cattuto et al., 2010; Olgui̇n et al., 2009). Note, however, that the statistics that we used to summarize the embedding of the student pairs into the larger network are only a sample of the wide range of statistics that are available, especially for directed events (e.g., degree statistics, reciprocity, etc.).

Throughout our analyses, we showcased some aspects of the flexibility of the REM framework to incorporate past events. In typical relational event models, it is assumed that all past events are equally influencing the probability of next events. Some studies have relaxed this assumption. For example, it is reasonable to expect that the influence of past events decreases as time goes by, giving a higher weight to more recent events (Brandes et al., 2009; Mulder and Leenders, 2019). In our analyses with the moving window approach, we captured the decrease in the importance of events over time by including only the most recent events in the endogenous predictors. Other factors may influence the weight of past events as well. For example, the events in the CONNECT data differed in duration and number of actors involved, two factors that are likely to influence how important an event is for predicting future events. Therefore, we defined a detailed inertia measure that accounts for these factors. In doing so, we deviated from the more conventional measure of inertia that counts the number of past events for a student pair (*i*, *j*) in the risk set at time *t*, weighing each event equally. Table S1 in the Supplementary Materials^3^ shows the results of additional analyses in which we compare our measure of inertia to a more conventional measure. These results indicate that our weighted inertia measure outperforms the conventional measure in terms of model fit and prediction performance. These conclusions encourage future research into how the influence of past events is best integrated in, for example, an inertia statistic.

### Neighboring approaches

Depending on the nature of the research question and the structure of the available data, a number of related statistical approaches may be appropriate for the data analysis. Current statistical approaches that are used in psychological research for the analysis of longitudinal social interaction (network) data (e.g., see ; Nestler et al., 2015, for an overview) include the social relations model (SRM; ; Kenny & La Voie, 1984), continuous-time models (Voelkle et al., 2012), (separable) temporal exponential random graph models ((S)TERGMs; ; Hanneke et al., 2010; Krivitsky & Handcock, 2014; Lusher et al., 2013; Robins & Pattison, 2001) and stochastic actor-oriented models (SAOMs or SIENA models; Snijders et al., 2010). In comparison, relational event models (a) can take into account quite complex higher-order network dependencies, and (b) are especially suited for longitudinal social interaction data observed on a fine-grained time scale (e.g., with real-time timestamps).

Most of the alternative models are best suited to analyze network dependencies that do not go beyond the dyad and/or work best with panel data (in which social network data is collected at multiple time points). While (S)TERGMs and SAOMs allow accounting for network dependencies in a way that is similar to relational event models, they would require the continuous-time data to be aggregated to a set of repeated networks; this creates artificial network observations and disposes of information on the time and order of the events in the aggregated sequences. Consequently, all information about social interaction dynamics is disregarded in the analysis. Instead, relational event models enable researchers to study how the history of interaction influences the probability for future social interaction, thereby continuously updating the past. Thus, when we have continuous-time interaction data (or interaction data in which the order of relational events is known), relational event models allows the researcher to perform the most detailed analysis, utilizing the full information available inside the data.^4^

Whereas the REM parameterizes the rate of interaction for a dyad, it is also possible to separate the dyadic activity by, first, modeling *who* is going to be the sender of the next relational event (including *when* the event is going to take place) and then, second, select who is going to be the receiver, *given who the sender is*. This approach is called the dynamic network actor model (DyNAM) (Stadtfeld et al., 2017; Stadtfeld and Block, 2017). The DyNAM framework is in many aspects similar to the REM framework, with the difference that it models social interaction in a two-step approach. Since the two modeling steps are conditionally independent, two sets of model parameters can be estimated for the two different models. The DyNAM focuses on who is a likely receiver for a given sender, weighting every potential event relative to the other available choices for the active individual (Stadtfeld & Block, 2017). Alternatively, the REM focuses on which event of all possible events is likely to occur next, weighting each potential event relative to all possible events (Stadtfeld & Block, 2017). Hence, statistically, the differences between these two major statistical frameworks for modeling relational event history data result in a somewhat different interpretation of the estimated model parameters. Substantively, the DyNAM considers interactions to be driven by the sender, whereas the REM considers interactions to be driven by both parties involved alike. Both models can be setup to yield similar results to the other model, although some types of interaction and some drivers of interaction fit more naturally with one approach or the other. In this paper, we presented how the REM can be used to study how interaction develops among a new group of students. However, note that many of the ideas and approaches in this paper can also be used if a DyNAM model is chosen.

### Conclusions

This paper provided an introduction to the REM framework for quantitative psychological researchers who are interested in social interaction dynamics. Relational event modeling is currently an active field of study, constantly increasing its significance for the study of social interactions across research domains. With the tools provided in this paper, we hope that we can stimulate the application of the REM framework within diverse fields of psychological research to help developing a more precise and fine-grained understanding of social interaction dynamics and how they evolve in continuous time. Most of the well-developed and influential theories of human and interpersonal behavior are quiet about the speed by which effects occur. Similarly, they tend not to inform a researcher about how they develop (suddenly, gradually, perhaps plateauing along the way?), about how long effects are likely to last, of about how they wane (suddenly, slowly, etc.). Also, little is known whether the effect of one driver of interpersonal behavior occurs faster than another (let alone how much faster). Approaches like the relational event model enable researchers to get a better idea of these things. This allows us to further develop and refine existing theories and develop new ones, being informed by the type of empirical findings these models can provide. Considering that the world is not a static place and very few drivers of (interpersonal) behavior can be expected to kick in immediately and last indefinitely, we believe that much academic progress can be made by studying the temporal development/behavior of effects. Of course, researchers do not always have access to ordered or time-stamped interaction data, but technological developments do assist in making such data increasingly available. In this case, we believe that statistical models like the relational event model, provide researchers with the tools to achieve important theoretical and empirical progress in their quest to further understand our changing world.

## Open practices statement

The CONNECT data used in the analyses in this paper as well as the code for all analyses can be found at https://osf.io/xjbm7/. None of the analyses were preregistered.

## Appendix A: Script I: Basic REM

# Install required R packages# install.packages("relevent")# remotes::install_github("TilburgNetworkGroup/remify")# remotes::install_github("TilburgNetworkGroup/remstats", ref = "V2.0.1")# Load the packageslibrary(remify)library(remstats)library(relevent)# Load pre-processed data objectsload("CONNECT.RData")# Specify the statistics to be computedstats <- ~ (minimum("extraversion") + maximum("extraversion") + minimum("agreeableness") + maximum("agreeableness")): (inertia(scaling = "std") + spUnique(scaling = "std")) + tie(both_male, "both_male") + difference("sex") + tie(both_old, "both_old") + difference("age") + event("group") + event("weekend")# Call remstats to compute the statisticsout <- remstats(tie_effects = stats, edgelist= eventseq, attributes = info, actors =info$id, directed = FALSE, origin = 0)# Extract the relevant objects from theoutput statistics <- out$statisticsevls <- out$evls# Induce a small time difference betweendyads interacting in groupsrehObject <- reh(eventseq, actors = info$id,directed = FALSE, origin = 0)evls[,2] <- cumsum(rehObject\InEq{$intereventTime)# Get the parameter estimates for the fivemodelsfit0 <- rem(evls, array(statistics[,,1], dim = c(dim(statistics[,,1]), 1)), timing = "interval", estimator = "MLE")fit1 <- rem(evls, statistics[,,c(1:5)], timing = "interval", estimator = "MLE")fit2 <- rem(evls, statistics[,,1:7], timing = "interval", estimator = "MLE")fit3 <- rem(evls, statistics[,,1:13], timing = "interval", estimator = "MLE")fit4 <- rem(evls, statistics, timing = "interval", estimator = "MLE")

## Appendix B: Script II: moving window REM

# Load the packages (see for installationscript I)library(remify)library(remstats)library(relevent)# Load data objectsload("CONNECT.RData")# Define the windowswindows <- data.frame( begin = c(0, seq(from = 961, to = 28321, by = 1440)), end = seq(from = 3840, to = 32640, by = 1440))# Find the event indices for when thewindows start and stop windows$}start <-apply(windows, 1, function(x) { start <- min(which(eventseq$time >as.numeric(x[1]))) ifelse(start == 0, 1, start)})windows$stop <- apply(windows, 1, function(x) { stop <- min(which(eventseq$time >as.numeric(x[2])))-1 ifelse(stop == Inf, nrow(eventseq), stop)})# Specify the statistics to be computedstats <- ~ (minimum("extraversion") +maximum("extraversion") +minimum("agreeableness") +maximum("agreeableness")): (inertia(scaling = "std") +spUnique(scaling = "std")) + tie(both_male, "both_male") +difference("sex") + tie(both_old,"both_old") + difference("age") + event("group")# Run a for loop over the windows to getfor each window the parameter estimatesfit0 <- fit1 <- fit2 <- fit3 <- fit4 <-list() # saving spaceevlsList <- statsList <- list() # savingspacefor(i in 1:nrow(windows)) { # Call remstats to compute the statistics out <- remstats(tie_effects = stats, edgelist = eventseq, directed = FALSE, actors = info$id, attributes = info, memory = "window", memory_value = 4319, start = windows$start[i], stop = windows$stop[i]) # Extract the relevant objects from the output statistics <- out$ statistics evls <- out$evls # Induce a small time difference between the dyads interacting in groups rehObject <- reh(eventseq[windows$start [i]:windows$stop[i],], actors = info$id, directed = FALSE, origin = windows$begin[i]) evls[,2] <- cumsum(rehObject\InEq{$intereventTime) # Get the parameter estimates for the five models fit0[[i]] <- rem(evls, array(statistics[,,1], dim = c(dim(statistics[,,1]), 1)), timing = "interval", estimator = "MLE") fit1[[i]] <- rem(evls, statistics[,,1:5)], timing = "interval", estimator = "MLE") fit2[[i]] <- rem(evls, statistics[,,1:7], timing = "interval", estimator = "MLE") fit3[[i]] <- rem(evls, statistics[,,1:12], timing = "interval", estimator = "MLE") fit4[[i]] <- rem(evls, statistics, timing = "interval", estimator = "MLE")}

## Appendix C: Script III: REM with event types

# Load the packages (see for installationscript I)library(remify)library(remstats)library(relevent)# Load data objectsload("CONNECT.RData")# Define the windowswindows <- data.frame( begin = c(0, seq(from = 961, to = 28321, by = 1440)), end = seq(from = 3840, to =32640, by = 1440))# Find the event indices for when thewindows start and stopwindows$}start <-apply(windows, 1, function(x) { start <- min(which(eventseq$time >as.numeric(x[1])))-1 ifelse(start == 0, 1, start)})windows$stop <- apply(windows, 1, function(x){ stop <- min(which(eventseq$time >as.numeric(x[2])))-1 ifelse(stop == Inf, nrow(eventseq), stop)})# Define the type columncolnames(eventseq)[4] <- "type"# Specify the statistics to be computedstats <- ~ inertia(scaling = "std") + spUnique(scaling = "std") + minimum("extraversion")+ maximum("extraversion") + minimum("agreeableness") +maximum("agreeableness") + tie(both_male, "both_male") +difference("sex") + tie(both_old,"both_old") + difference("age") + event("group") + FEtype() + inertia(scaling = "std", consider_type= TRUE) + spUnique(scaling = "std",consider_type = TRUE) + FEtype() : (minimum("extraversion") +maximum("extraversion") +minimum("agreeableness") +maximum("agreeableness"))# Run a for loop over the windows to get foreach window the parameter estimates formodels 3, 5 and 6fit3 <- fit5 <- fit6 <- list() # saving spacefor(i in 1:nrow(windows)) { # Call remstats to compute the statistics out <- remstats(tie_effects = stats, edgelist = eventseq, directed = FALSE, actors = info$id, attributes = info, memory = "window", memory_value = 4319, start = windows$start[i], stop = windows$stop[i]) # Extract the relevant objects from the output statistics <- out$statistics evls <- out$evls # Induce a small time difference between the dyads interacting in groups rehObject <- reh(eventseq[windows$start [i]:windows$stop[i],], actors = info$id,directed = FALSE, origin = windows$begin[i]) evls[,2] <-cumsum(rehObject$intereventTime) # Get the parameter estimates fit3[[i]] <- rem(evls, statistics[,,c(1:12)], timing = "interval", estimator= "MLE")fit5[[i]] <- rem(evls,statistics[,,1:15], timing = "interval",estimator = "MLE")fit6[[i]] <- rem(evls,statistics, timing = "interval",estimator = "MLE")}

## Footnotes

1 A sensitivity analysis with three different randomizations found no meaningful differences in results.

2 The remstats package for R can be downloaded from https://github.com/TilburgNetworkGroup/remstats.

3 https://osf.io/xjbm7/

4 For a detailed overview of the differences between the REM, (S)TERGMs and SAOMs, we refer the interested reader to Quintane et al., (2014).
