# Author Correction: Discovering trends of social interaction behavior over time: An introduction to relational event modeling

**DOI:** 10.3758/s13428-024-02423-2

**Published:** 2024-05-06

**Authors:** Marlyne Meijerink-Bosman, Mitja Back, Katharina Geukes, Roger Leenders, Joris Mulder

**Affiliations:** 1 Department of Methodology & Statistics, Tilburg University, Warandelaan 2, 5037 AB Tilburg, The Netherlands; 2 Department of Psychology, University of Münster, Münster, Germany; 3 Department of Organization Studies, Tilburg University, Tilburg, The Netherlands; 4 Jheronimus Academy of Data Science, ’s-Hertogenbosch, The Netherlands

**Author Correction: Behavior Research Methods (2022) 55:997-1023**

10.3758/s13428-022-01821-8

The original online version of this article was revised. Funding note has been updated to say: This research was supported by the Netherlands Organization for Scientific Research (NWO) Grant to JM and MMB (452-17-006), as well as the ERC Starting Grant 'TIMEISNOW' to JM and RL (758791).

